# Little effects on soil organic matter chemistry of density fractions after seven years of forest soil warming

**DOI:** 10.1016/j.soilbio.2016.09.003

**Published:** 2016-12

**Authors:** Jörg Schnecker, Werner Borken, Andreas Schindlbacher, Wolfgang Wanek

**Affiliations:** a Department of Natural Resources and the Environment, University of New Hampshire, Durham, NH, USA; b Department of Microbiology and Ecosystem Science, University of Vienna, Vienna, Austria; c Department of Soil Ecology, University of Bayreuth, Bayreuth, Germany; d Department of Forest Ecology, Federal Research and Training Centre for Forests, Natural Hazards and Landscape – BFW, Vienna, Austria

**Keywords:** Warming, Climate change, Density fractionation, Organic matter chemistry

## Abstract

Rising temperatures enhance microbial decomposition of soil organic matter (SOM) and thereby increase the soil CO_2_ efflux. Elevated decomposition rates might differently affect distinct SOM pools, depending on their stability and accessibility. Soil fractions derived from density fractionation have been suggested to represent SOM pools with different turnover times and stability against microbial decomposition.

To investigate the effect of soil warming on functionally different soil organic matter pools, we here investigated the chemical and isotopic composition of bulk soil and three density fractions (free particulate organic matter, fPOM; occluded particulate organic matter, oPOM; and mineral associated organic matter, MaOM) of a C-rich soil from a long-term warming experiment in a spruce forest in the Austrian Alps. At the time of sampling, the soil in this experiment had been warmed during the snow-free period for seven consecutive years. During that time no thermal adaptation of the microbial community could be identified and CO_2_ release from the soil continued to be elevated by the warming treatment. Our results, which included organic carbon content, total nitrogen content, δ^13^C, Δ^14^C, δ^15^N and the chemical composition, identified by pyrolysis-GC/MS, showed no significant differences in bulk soil between warming treatment and control. Surprisingly, the differences in the three density fractions were mostly small and the direction of warming induced change was variable with fraction and soil depth. Warming led to reduced N content in topsoil oPOM and subsoil fPOM and to reduced relative abundance of N-bearing compounds in subsoil MaOM. Further, warming increased the δ^13^C of MaOM at both sampling depths, reduced the relative abundance of carbohydrates while it increased the relative abundance of lignins in subsoil oPOM. As the size of the functionally different SOM pools did not significantly change, we assume that the few and small modifications in SOM chemistry result from an interplay of enhanced microbial decomposition of SOM and increased root litter input in the warmed plots. Overall, stable functional SOM pool sizes indicate that soil warming had similarly affected easily decomposable and stabilized SOM of this C-rich forest soil.

## 1 Introduction

Mean annual temperatures in the alpine region are expected to increase by more than 4 °C by the end of this century (Gobiet et al., 2014). Higher temperatures lead to enhanced microbial activity and higher CO_2_ release from soil, imposing the probability of warming induced reductions in soil C stocks (Cox et al., 2000; Lloyd and Taylor, 1994). While increased CO_2_ production can be detected in long-term soil warming experiments (Melillo et al., 2011; Reth et al., 2009; Schindlbacher et al., 2009), changes in the soil organic matter stocks and especially changes in soil organic matter chemistry, however, might not be readily visible. Changes in the chemical composition of SOM, e.g. a depletion of easily available carbon C-forms have been made responsible for a decline in the response of CO_2_ production with warming (Kirschbaum, 2004; Melillo et al., 2002). Depletion of easily available C-forms and a higher microbial activity might also force the microbes to alter their substrate source utilization pattern towards more stable SOM pools (Frey et al., 2013). This might not only change the size of these pools but also the chemical composition. SOM chemistry of different pools might thus be a crucial component to understand and estimate the response of decomposition processes to warming. However, little is known about the effect of warming on different functional pools of SOM.

Soil fractions derived from density fractionation have been argued to represent different pools of SOM with varying turnover times, accessibility by microbes and thus stability (Crow et al., 2007; Schulze et al., 2009; von Lützow et al., 2007). Plant litter enters the SOM pool as free particulate organic matter (fPOM). fPOM is characterized by fast turnover times and low stability. It usually consists of easily degradable plant components such as cellulose, starch and other carbohydrates (von Lützow et al., 2007), with smaller contributions by lignin, cutin and other compounds that are less decomposable. Products of enzymatic decomposition of fPOM as well as soluble litter components can be taken up by microbes or can become part of the mineral associated organic matter (MaOM), along with the now microbially transformed compounds. Usually the largest percentage of SOM is mineral-associated; this fraction is considered to have the longest time of up to >1000 years, depending on the minerals present (Schulze et al., 2009). Mineral association also provides the greatest protection from microbial decomposition (Baisden et al., 2002; Poirier et al., 2005) and MaOM is sometimes even referred to as a passive pool (e.g. Gaudinski et al., 2000; Schulze et al., 2009). Both fPOM and MaOM can be incorporated in aggregates (Poirier et al., 2005). While in aggregates POM is only slowly transformed and it is older and more stable than fPOM (von Lützow et al., 2007). Based on their properties with respect to stability and persistence, the size, turnover time, and chemical composition of these SOM pools, but also the transformations from one fraction to another might be affected by soil warming in different ways.

To study the effect of soil warming on the chemical composition of different soil organic matter fractions, we took advantage of a soil warming experiment in Achenkirch/Austria where soil had been warmed by 4 °C during the snow-free seasons from 2005 until the current study in 2012. In contrast to other long-term soil warming experiments, the positive response of soil respiration to warming (~40% increase) remained stable throughout the seven years of warming (Schindlbacher et al., 2012). The sustained enhancement of soil respiration suggests that substrate depletion did not play a major role in this C-rich soil so far. Additionally the microbial community in these soils did not show strong signs of physiological adaptation to the persisting higher temperatures (Schindlbacher et al., 2011, 2015; Kuffner et al., 2012).

Based on the suggested different properties of the three density fractions (fPOM, oPOM and MaOM), we hypothesized that soil warming led to (I) a reduction of plant derived compounds such as carbohydrates and lignin (Gleixner et al., 2002), especially in the fPOM fraction and (II) increased microbial transformation of the MaOM fraction indicated by lower C:N ratios, higher δ^13^C and δ^15^N (Six et al., 2001; Wallander et al., 2004), as well as increases in N-bearing compound contents (Gleixner et al., 2002).

To that end, we determined organic C (OC) and total N (TN) contents, δ^12^C, δ^15^N and Δ^14^C values of the bulk soil and of the three individual density fractions, fPOM, oPOM and MaOM. In addition we analyzed the samples with pyrolysis gas chromatography-mass spectrometry (Py-GC/MS) to determine the effect of warming on the macromolecular composition of the different SOM pools, including contents of lignin, carbohydrates and the contents of N-bearing compounds.

## 2 Material and methods

### 2.1 Site description and soil sampling

Soils for this study where sampled at a long-term soil warming experiment located near Achenkirch, Austria (47° 34′ 50′′ N; 11° 38′ 21′′ E). The experimental site is situated on a north-north-east slope of a mountain in the Northern Limestone Alps at 910 m a.s.l. The snow-free period in this area lasts from April/May to November/December. Local mean annual air temperature and precipitation were 6.9 °C and 1506 mm (1992–2012), respectively (Achenkirch village; ~7 km away at similar altitude; data from Zentralanstalt für Meteorologie und Geodynamik (ZAMG)). The ~130-years old forest is dominated by Norway spruce (*Picea abies*), with interspersed European beech (*Fagus sylvatica*) and silver fir (*Abies alba*). The soils are a mosaic of shallow Chromic Cambisols and Rendzic Leptosols. The bedrock is dolomite. Soils are characterized by high carbonate content and near neutral pH. Organic C stocks were estimated to be ~10 t ha^−1^ in the organic layer and ~120 t ha^−1^ in the mineral soil (Schindlbacher et al., 2009).

The warming experiment was set up in 2004 (Schindlbacher et al., 2009). Three pairs of control and warmed plots were established. Warmed plots are heated by resistance heating cables (0.4 cm diameter, TECUTE – 0.18 Ohm/m/UV, Etherma, Austria). The cables were buried in 3 cm deep slots and had a spacing of 7–8 cm. The soil temperature of each warmed subplot was kept 4 °C above that of the adjacent control subplot during the snow free-seasons, starting in spring 2005. At control plots (n = 3) cables were inserted but not heated.

Sampling took place in October 2012. Plots were sampled using a soil corer with a diameter of 5 cm. From each plot 10 cores were taken and bulked according to their sampling depth. Samples were air-dried at 60 °C before further analysis.

### 2.2 Density fractionation of soil

Sieved (<2 mm) and air-dried (60 °C) mineral soil samples from 0–10 and 10–20 cm of each plot were fractionated by density separation. For this purpose, the samples were dispersed in sodium polytungstate solution (SPT, Sometu, Berlin, Germany) at densities of 1.6 g cm^−3^ and 2.0 g cm^−3^ using a similar procedure as described by John et al. (2005). First, 10 g soil and 40 ml of SPT with a density of 1.6 g cm^−3^ were gently shaken. After sedimentation, the solution was centrifuged at 5085 *g* for 1 h (Varifuge 3.2RS). The supernatant was filtered through 0.45 μm pre-washed cellulose-acetate-filter (Schleicher & Schuell, Germany) and the fPOM fraction <1.6 g cm^−3^ was washed with 200 ml deionized water. Then the pellet was dispersed with 2.0 g cm^−3^ SPT and 10 glass beads and was shaken for 16 h at 60 rpm and centrifuged at 5085 g for 1 h. The supernatant with particles <2.0 g cm^−3^ (oPOM) was washed with 200 ml deionized water and filtered through 0.45 μm cellulose-acetate filters. The pellet contained the mineral associated organic matter fraction >2.0 g cm^−3^ (MaOM). To remove the SPT salt, the pellet was washed three times with deionized water. Thereafter, carbonate was removed from density fractions and bulk soil by stepwise addition of ultra-pure hydrochloric acid (HCl) until a constant pH value of 2.0 was reached during incubation at 60 °C over 2–3 weeks. Again, the soil samples were stepwise washed with deionized water to remove HCl down to an electric conductivity of 1 mS m^−1^ in the supernatant. Removal of SPT salt by washing and treatment with HCl to remove carbonates caused a loss of dissolved organic carbon (DOC) in all density fractions, ranging between 2 and 8% of total bulk soil C. The fPOM, oPOM, MaOM fractions were freeze-dried and then finely ground with a ball mill for C, N, and isotope analyses.

### 2.3 Organic C, total N, δ^13^C, and δ^15^N

Organic C (OC) content, TN content, δ^13^C, and δ^15^N of bulk mineral soil and density fractions were measured on dried and finely ground samples using elemental analyzer-isotope ratio mass spectrometry (EA-IRMS; CE Instrument EA 1110 elemental analyzer, coupled to a Finnigan MAT DeltaPlus IRMS with a Finnigan MAT ConFlo II Interface). Reference gas (high purity N_2_ and CO_2_) was calibrated to the atmospheric air N_2_ (at-air) and the Vienna-Pee Dee Belemnite (V-PDB) standards using certified reference materials provided by the International Atomic Energy Agency (Vienna, Austria). The natural abundance of ^15^N and ^13^C was calculated as follows:

$$\delta^{15}\text{N}[‰\;\text{vs}.\;\text{at}-\text{air}]=\left({\text{R}}_{\text{sample}}/{\text{R}}_{\text{standard}}-1\right)\times 1000$$

$$\delta^{13}\text{C}[‰\;\text{vs}.\;\text{V}-\text{PDB}]=\left({\text{R}}_{\text{sample}}/{\text{R}}_{\text{standard}}-1\right)\times 1000$$

where R denotes the ratio of ^15^N/^14^N or ^13^C/^12^C. The standard deviation of repeated measurements of a laboratory standard was better than 0.10‰ for both isotope signatures.

Prior to analysis carbonate was removed from bulk soil samples by repeated acidification with 2 M HCl. Dissolved organic C released during the acidification procedure was not lost as the acidic supernatant was not removed but re-dried into the soil residue at 85 °C before re-homogenization in a ball mill.

### 2.4 Radiocarbon analysis

Radiocarbon signatures of bulk soil and density fractions were determined by accelerator mass spectrometry (AMS). Subsamples of 1 mg C were combusted in 6 mm sealed quartz tubes with 60 mg CuO oxidizer and 1 cm silver wire for 2 h at 900 °C. The resulting CO_2_ was purified from water and non-condensable compounds. Afterwards, CO_2_ was reduced to graphite using the zinc reduction method where TiH_2_ and Zn with Fe act as catalysts at 550 °C for 7.5 h (Xu et al., 2007). All preparations took place at the Department of Soil Ecology at the University of Bayreuth. The graphite targets were analyzed by the Keck-CCAMS facility of the University of California, Irvine, USA, with a precision of 2–3‰. Radiocarbon data are expressed as Δ^14^C (‰ deviation was from the ^14^C/^12^C ratio of oxalic acid standard in 1950). The samples have been corrected to a δ^13^C value of −25‰ to account for any mass dependent fractionation effects (Stuvier and Polach, 1977).

### 2.5 Pyrolysis gas chromatography-mass spectrometry

To determine the chemical composition of soil organic matter, we used pyrolysis gas chromatography and mass spectrometry (Py-GC/MS). Samples for Py-GC/MS had not been treated with HCl before analysis. The analysis was performed with a Pyroprobe 5250 pyrolysis system (CDS Analytical) coupled to a Thermo Trace gas chromatograph and an ISQ MS detector (both Thermo Scientific). The GC was equipped with a carbowax column (Supelcowax 10, Sigma-Aldrich). Approximately 1 mg of the dried and finely ground sample was heated at a rate of 20 °C/s and kept at 600 °C for 20 s in a helium atmosphere. GC oven temperature was constant at 50 °C for 2 min, followed by an increase of 7 °C/min to a final temperature of 260 °C, which was held for 15 min. The MS detector was set for electron ionization at 70 eV in the scanning mode (m/z 20–300).

Peaks were assigned based on NIST 05 MS library after comparison with measured reference materials. 86 peaks were identified and selected for integration either because of their abundance or diagnostic value. Relative peak areas are stated as % of the sum of all integrated peaks.

### 2.6 Statistics

All statistical analysis were performed in R 3.0.2 (R Development Core Team, 2013). Data were tested for normality with the Shapiro-Wilk test and for homogeneity of variances with the Bartlett's test. Depending on the results of these tests we used paired two-sample comparison tests (*t*-test, Welch test or Mann-Whitney-U test) to determine differences between treatments. If data for ANOVA did not meet the criteria of normality and homoscedasticity data were log transformed or rank-normalized. Differences between control and warming treatment were calculated by subtracting the values of the warmed plots from those of the respective control plots. The so obtained Δ-values for total C, total N, C:N, δ^13^C, Δ^14^C, δ^15^N were used in correlations with each other. Residuals of the correlations were tested for normality and rank-normalized when necessary. To obtain a chemical fingerprint of the individual fractions, we calculated Euclidean distance matrixes from 86 individual substances detected with Py-GC/MS. We used these matrixes to create Nonmetric Multidimensional Scaling (NMDS) plots. A list of the individual compounds, their species scores and the mean species scores of the compound classes is provided in Table S1. To identify differences between fractions, sampling depth, treatment and their interactions we used Permutational Multivariate Analysis of Variance Using Distance Matrices (ADONIS). This analysis is implemented in the R-package vegan (Oksanen et al., 2013). Mantel tests were used to correlate the obtained distance matrix of all samples and individual density fractions with the other measured parameters (OC, TN, C:N ratios, δ^13^C, Δ^14^C, δ^15^N).

## 3 Results

### 3.1 Density fraction yield and composition

**Fig. 1:**
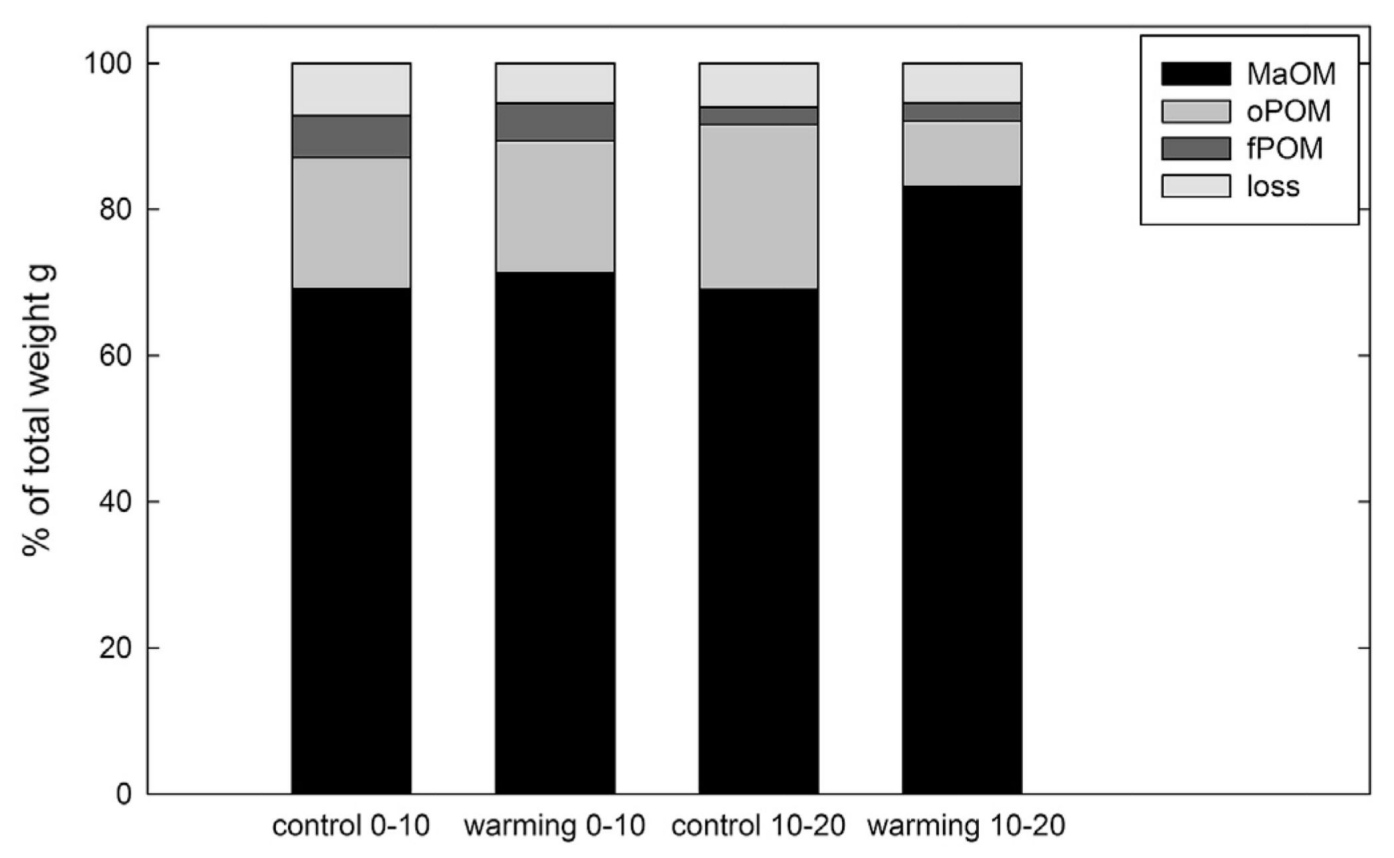
Weight distribution amongst the three different fractions in control and warming lots at 0–10 cm soil depth and 10–20 cm soil depth. No warming induced significant differences could be found.

The total amount of soil recovered after fractionation was 93.1 ± 1.6% in 0–10 cm sampling depth and 94.3 ± 0.5% in 10–20 cm sampling depth. Total C recovered was 92.7 ± 5.5% in the upper soil compartment and 98.3 ± 6.1% in the lower soil compartment. The losses of material and C arise partly from the density fractionation which removes salt extractable material but also from the treatment with acid afterwards to remove carbonates. The distribution of weight and OC among the fractions was similar in the control and warming plots from 0 to 10 cm and the samples from 10 to 20 cm from the control plots (Fig. 1, Tables 1 and 2). In samples from warming plots from a sampling depth of 10–20 cm, oPOM only contributed 9% of the weight and 23.7% of total OC while in the comparable control soils it was 22.6% in weight and 51.4% in OC. These differences were, however, not significant.

### 3.2 Chemical and isotopic composition of bulk soil and soil fractions

Chemical and isotopic composition of bulk soil and the individual fractions is summarized in Table 1 (sampling depth of 0–10 cm) and Table 2 (sampling depth of 10–20 cm). Relative peak areas of individual substances obtained from Py-GC/MS were grouped in the categories carbohydrates, lipids and waxes, N-bearing compounds, aromatic compounds, lignin and phenolic substances. Most investigated parameters, with the exception of the relative abundance of carbohydrates, and lipids and waxes were significantly different between fractions (Table 3). Δ^14^C, δ^15^N and aromatic compounds showed significant differences between depth and over all fractions, and at both sampling depths δ^13^C and the relative abundance of lignin showed a significant effect of warming. Bulk soil from control plots and warming plots did not show any significant differences in any of the measured parameters (Tables 1 and 2). Warming induced changes in the individual fractions were sparse and small. In the upper soil compartment (0–10 cm) N content in the oPOM fraction and δ^13^C values in MaOM fraction significantly decreased in warming plots (Table 1). In the lower soil compartment (10–20 cm) N content of fPOM and again δ^13^C values of MaOM were significantly lower in the warming plots (Table 2). In addition, warming caused a lower relative content of carbohydrates and relatively more lignin compounds in lower soil oPOM. At 10–20 cm soil depth MaOM also showed a significant decrease in N-bearing compounds (Table 2). Warming induced changes, calculated by subtracting the value for warmed plots from the respective control value and expressed as D-values, showed significant relations to each other (Table S2). We found significant correlations between changes in total C and changes in total N and significant correlations between ΔC:N, Δδ^13^C, and ΔΔ^14^C. Moreover, across the decomposition continuum from roots to fPOM, oPOM and MaOM decreases in C:N were related to increases in δ^13^C, δ^15^N and to decreases in Δ^14^C (Figs. S1 and S2).

### 3.3 ^14^C signatures of soil fractions

Radiocarbon signatures of bulk soil and corresponding density fractions from 0 to 10 cm depth indicate predominately young, modern C (Table 1). The proportion of modern C decreases from fPOM to MaOM though there are no consistent changes towards more or less modern C in the density fractions from heated plots. Although not statistically significant, higher ^14^C signatures of the oPOM and MaOM fractions may result from enhanced loss of old post-bomb C and from increased incorporation of young organic matter by soil warming.

**Fig. 2:**
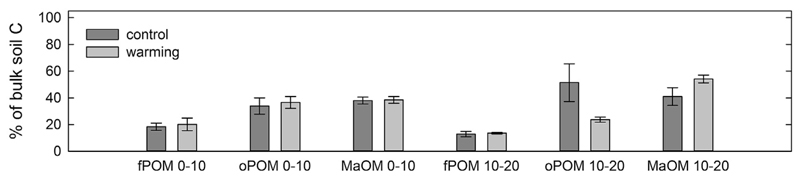
Distribution of total bulk soil C into the three fractions (fPOM, oPOM, and MaOM) at 0–10 cm soil depth and 10–20 cm soil depth. Control plots are in dark grey and warming plots are in light grey. No warming induced significant differences could be found (n = 3).

Bulk soil and density fractions from 10 to 20 cm depth tended to consist of older C with radiocarbon signatures of –48.7 to 61.1‰ (Table 3). In agreement with the 0–10 cm depth, the C of the oPOM and MaOM fractions was, although not statistically significantly, younger in the heated plots than in the control plots. As most C at this depth is stored in these fractions (Fig. 2), the Δ^14^C signature of bulk soil indicates overall younger C at 10–20 cm in the warmed plots. The fPOM fractions exhibited similar Δ^14^C signatures in the control and heated plots.

### 3.4 Chemical fingerprint of soil fractions

**Fig. 3:**
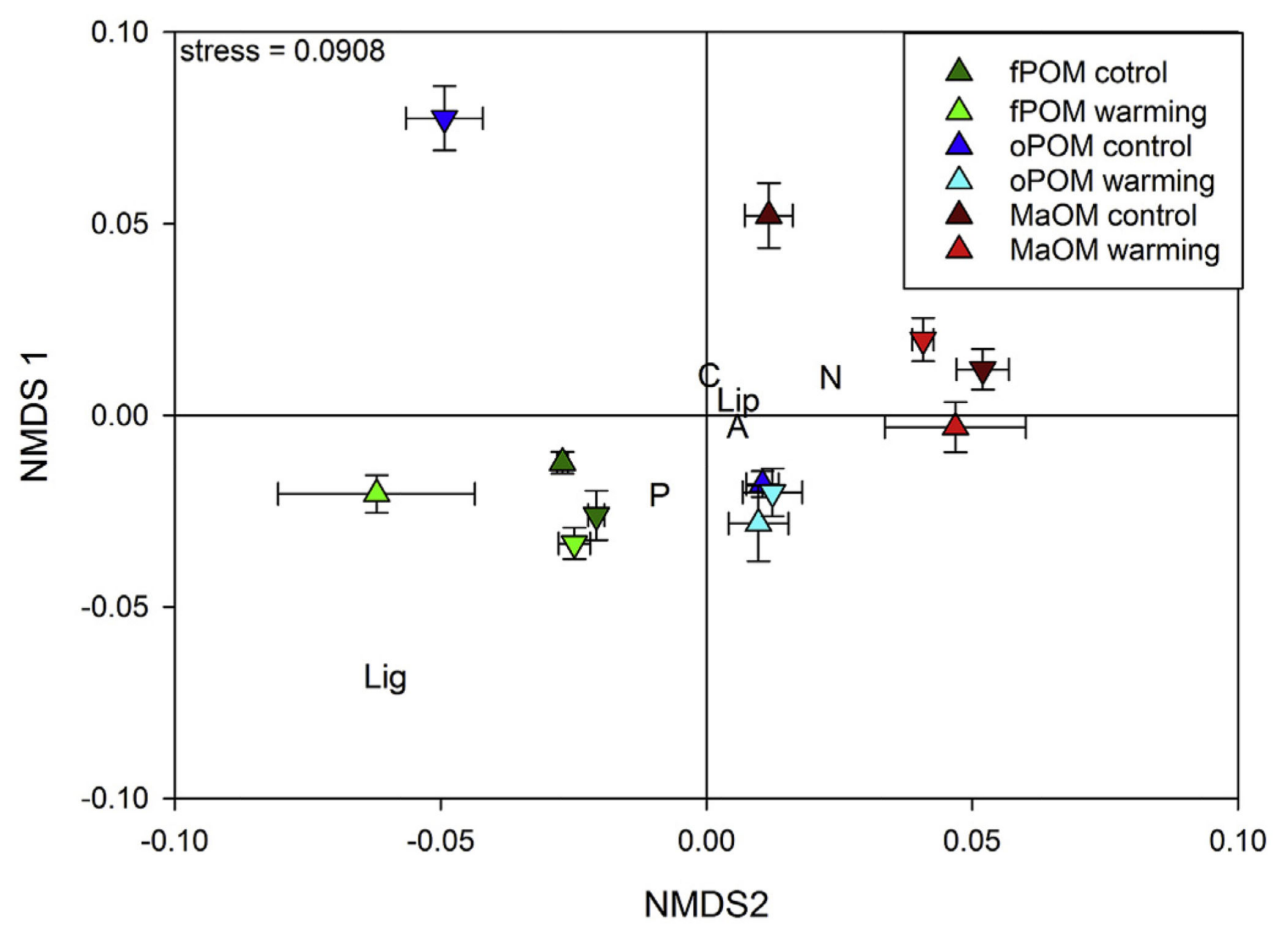
Chemical fingerprint of density fractions visualized as NMDS plot of chemical composition based on a Euclidean distance matrix of 86 individual substances detected by Py-GC/MS. Symbols are mean values the individual fraction (fPOM is green, oPOM is blue, and MaOM is red) from replicated individual treatment plots (dark colors indicate control and light colors indicate warming) sampled at 0–10 cm (pyramids) and 10–20 cm (inverted pyramids). The letters are mean species scores of the projected individual peaks according to their compound classes (C are carbohydrates, N are N-bearing compounds, Lip are lipids and waxes, A are aromatic compounds, P are phenolic compounds and Lig are Lignin-derived compounds). Results from Permutational Multivariate Analysis of Variance Using Distance Matrices (ADONIS) accompanying this graph can be found in Table 3. (For interpretation of the references to colour in this figure legend, the reader is referred to the web version of this article.)

The chemical fingerprint of the individual density fractions, obtained from a Euclidean distance matrix using 86 individual Py-GC/MS products (Fig. 3), shows a clear separation of the three density fractions. This is supported by the results of the ADONIS (Table 3) which shows a significant difference between fractions. The warming treatment significantly changed the chemical fingerprint. However the direction of these changes varied between fractions and depths which is supported by the statistically significant interaction terms of the ADONIS (Table 3). Warming caused a slight shift in the light fraction (fPOM) in the upper 0–10 cm while no change could be found at the lower sampling depth (Fig. 3). In the occluded light fraction (oPOM), warming shifted the chemical fingerprint of the samples from 10 to 20 cm in the direction of the samples from 0 to 10 cm soil depth. In MaOM the opposite happened and the chemical fingerprint of the topsoil samples were shifted in the direction of the subsoil samples. Mantel tests of the chemical fingerprint, calculated as distance matrix, with other soil chemical and isotopic parameters showed significant correlations of the whole matrix with all other parameters (OC, TN, C:N, δ^13^C, d^14^N and δ^15^N) reflecting the differences between the fractions (Table S3). Mantel tests of the individual fractions only showed significant correlations of the chemical fingerprint of oPOM with C:N ratio and Δ14C.

## 4 Discussion

After seven years of soil warming with a sustained increase in CO_2_ emission by 40% and no adaptation of microbial physiology (Schindlbacher et al., 2015), we found no significant changes in the size and chemical composition of the bulk soil. In contrast to our hypothesis, also the functionally different SOM pools isolated by density fractionation were not changed in size by warming. In addition, the chemical composition of these pools was only slightly altered and the changes did not show a consistent direction with soil warming.

Surprisingly, we could also not find a stronger warming effect in the topsoil (0–10 cm) than in the subsoil (10–20 cm) although the heating cables were buried at 3 cm depth. While warming changed the chemical fingerprint of fPOM and MaOM (Fig. 3) and N content in oPOM and δ^13^C of MaOM in the topsoil (Table 1), it also altered the chemical fingerprint of oPOM (Fig. 3) and significantly changed N content of fPOM, δ^13^C and the relative amount of N-bearing compounds in MaOM, as well as the relative amount of carbohydrates and lignins in oPOM of the subsoil (Table 2).

One possible explanation for the few and small differences in the individual density fractions of warmed and control soils might be that the turnover of all fractions was increased similarly, assuming the transformation pathways also remained the same. Enhanced turnover of the individual fractions and faster exchange between them was suggested by Cheng et al. (2011) who found increased processing of litter and consequent incorporation in different soil fractions as a result of soil warming in a grassland system. While multiple studies have shown how different fractions individually react to higher temperatures (Leifeld and Fuhrer, 2005; Wagai et al., 2013; Benbi et al., 2014), warming induced changes might be different when these fractions remain combined as bulk soil during the warming (Swanston et al., 2002; Mueller et al., 2014). In the bulk soil, warming has not only direct effects on the individual fractions, but increased decomposition of fPOM for instance could lead to an increase of soluble compounds and ultimately replace organic matter in the MaOM fraction. This competitive sorbant displacement (Kleber et al., 2015) could thus lead to no net changes in the pool size of MaOM, but may change the chemical composition.

The potential scenario of an increased turnover of all fractions is also supported by the radiocarbon data (Tables 1 and 2). The modern ^14^C signatures of all density fractions from the topsoil as well as the fPOM and oPOM fractions from the subsoil indicate relatively young organic SOM pools in the control and warming plots. Hence, a large part of the total SOC stock is rather unstable and continuously replaced by large amounts of litter or microbially transformed compounds. Other temperate forest soils are characterized by negative ^14^C signatures in the subsoil or even in the topsoil (e.g., Gaudinski et al., 2000; Schulze et al., 2009). Constituting only a small fraction, the subsoil MaOM fraction had negative ^14^C signatures, was old (calendar age of 170–400 years) and more stable than the other density fractions. However, as ^14^C signatures were not significantly different between the treatments, we assume that all SOM pools contributed to the elevated CO_2_ efflux in the warming plots. Considering the high organic C contents of the Achenkirch soil, the C losses by warming might be hitherto too small for significant changes in specific SOM pools. Similar to our study, ^14^C signatures of respired CO_2_ indicated enhanced losses of years-to-decades old soil organic C by warming (Hopkins et al., 2012).

Our findings of few and small changes in SOM chemistry and isotopic composition is also in contrast with other studies investigating the effect of soil warming. After only four years of warming, Pisani et al. (2015) found a decrease in labile SOM components and signs for an accelerated degradation of lignin and cutin, and assigned these changes to a stimulation of decomposition by soil microbes. A different effect of soil warming on SOM was found by Feng et al. (2008). After 14 month of soil warming the authors of this study found an increase of leaf-cuticle-derived substances in a mixed temperate forest. At this study site, however, the litter C input into the soil exceeded the C loss by SOM decomposition. These two examples show that soil warming alters SOM chemistry through two mechanisms: enhanced microbial decomposition (Pisani et al., 2015) and increased plant litter input (Feng et al., 2008).

These two mechanisms may however change individual chemical and isotopic parameters in the exact opposite way. δ^13^C values of organic matter commonly increase with microbial transformation while more plant inputs decrease them (Six et al., 2001; Taylor et al., 2003; Wallander et al., 2004). Soil C:N ratios decrease when SOM is microbially transformed, while enhanced fresh plant litter inputs usually increases soil C:N ratios (Six et al., 2001; Sollins et al., 2009). Lignin content decreases with advancing decomposition but increases with litter input (Gleixner et al., 2002).

In our study, we found small but statistically significant changes in SOM chemistry that could be attributed to both enhanced microbial decomposition and increased litter input. Warming for instance changed the chemical fingerprint of topsoil MaOM to become more similar to that of subsoil MaOM (Fig. 3). Since subsoil MaOM can be presumed to be more decomposed and more microbially transformed (Sollins et al., 2009), that shift could be explained by enhanced microbial decomposition and higher SOM turnover induced by warming, which is evident for the study site by increased soil respiration (Schindlbacher et al., 2009). In contrast, the chemical fingerprint (Fig. 3) of oPOM from the subsoil (10–20 cm) became more similar to that of oPOM from the topsoil (0–10 cm). The changes in oPOM, are opposite to the direction that would be expected by enhanced microbial activity. The same is true for the lower δ^13^C values of MaOM in the warming plots (Tables 1 and 2), which is indicative of a higher contribution of fresh unprocessed plant material (Six et al., 2001). Also the higher relative contribution of lignin and lower contribution of carbohydrates to the subsoil oPOM in warming plots compared to control and the before mentioned shift of subsoil oPOM towards topsoil oPOM (Fig. 3), can be assigned to a higher incorporation of plant material into subsoil oPOM in warming plots (Gleixner et al., 2002). A stronger plant signal can derive from a higher input of leaf litter, but this was not found at our study site or a change in litter chemistry, which is possible but unlikely in this soil warming experiment. The stronger plant signal might also derive from an increased production of roots. While no increase in the fine root biomass (<2 mm) could be found at the study site, an increased fine root turnover of 10–40% was detected based on radiocarbon estimates and ingrowth-coring (Borken et al. unpublished), translating to enhanced fine root production and increased root detrital inputs to soils.

These indications of increased root litter input in our study might be related to the duration of the experiment. The radio-carbon ages of live fine roots vary between 3 and 18 years and increase with soil depth (Gaudinski et al., 2001; Joslin et al., 2006). Such longevities of fine roots especially in the subsoil might have only become visible after the seven years of soil warming in our study, but not in the study by Pisani et al. (2015) which with four years might have been too short to pick up a changed plant signal. We do not know whether a steady-state of root production and root mortality was already accomplished after seven years of warming. The longevity generally increases with root diameter (Joslin et al., 2006) and thus also more time is needed for reaching the steady state of fine root turnover.

Both, increased root detrital input and enhanced microbial decay of SOM could have been drivers for the warming induced differences we found. Increased root litter counteracted with what we would have expected from increased microbial activity and decomposition alone. And while warming induced changes in these factors (C:N, δ^13^C, Δ^14^C) were significantly correlated (Table S2) and followed the litter-to-SOM continuum, these two counteracting mechanisms of enhanced microbial decomposition and higher fine root litter production might have, in part, balanced each other out and diminished the gross effects that each of these mechanisms would have had alone.

In summary, our results show little effects of soil warming on the chemical and isotopic composition of bulk soil. Surprisingly we also found only few and little changes in the density fractions of topsoil and subsoil. As we also did not find any changes in the size of the different SOM pools, despite ongoing increased CO_2_ efflux in the warmed plots of the study site, we speculate that such a result could have two potential explanations: 1) Enhanced microbial decomposition affected all density fractions similarly and net changes in C pools were buffered by the large carbon stocks of the studied ecosystem. 2) Enhanced microbial decomposition is superimposed in part by increased input of fine root litter. While we found little effects of soil warming on SOM chemistry and isotopic composition of bulk soil and density fractions, consistent and long lasting changes could appear with prolonged soil warming and decreasing SOM stocks in the following years.

## Appendix A. Supplementary data

Supplementary data related to this article can be found at http://dx.doi.org/10.1016/j.soilbio.2016.09.003.

FigS1

FigS2

TabS1

TabS2

TabS3

## Figures and Tables

**Table 1:** SOM chemistry of control and warming plots at 0–10 cm soil depth (n = 3). Values for carbohydrates, lipids and waxes, N-bearing compounds, aromatic compounds, lignin and phenolic compounds are derived from Py-GC/MS and are given in relative peak area. Bold numbers and asterisks indicate a statistically significant difference between control and warming treatments (* P-value < 0.05, ** P-value < 0.01). The plus and minus signs show whether warming caused an increase or a decrease in the respective parameter.

0–10 cm soil depth	Bulk		fPOM		oPOM		MaOM	
	Control	Warming	Control	Warming	Control	Warming	Control	Warming
% of bulk soil weight			5.70 ± 1.34	5.15 ± 1.20	18.0 ± 5.18	18.1 ± 3.88	69.1 ± 5.79	71.3 ± 5.59
Organic C content (% dry weight)	8.68 ± 1.62	6.43 ± 0.69	28.4 ± 0.64	30.5 ± 0.50	17.3 ± 0.77	16.5 ± 0.17	4.82 ± 0.79	4.29 ± 0.57
Total N content (% dry weight)	0.59 ± 0.11	0.44 ± 0.05	1.01 ± 0.04	1.09 ± 0.05	**1.07 ± 0.02**	0.97 ± 0.03 –*	0.40 ± 0.06	0.36 ± 0.05
C:N ratio	14.6 ± 0.15	14.7 ± 0.30	28.1 ± 0.61	28.0 ± 0.78	16.1 ± 1.01	17.1 ± 0.56	11.9 ± 0.34	11.9 ± 0.07
δ^13^C	−25.9 ± 0.02	−26.2 ± 0.06	−26.8 ± 0.11	−26.7 ± 0.16	−26.4 ± 0.14	−26.7 ± 0.10	**−25.8 ± 0.08**	−26.0 ± 0.06 −*
Δ^14^C	49.8 ± 9.4	57.6 ± 4.7	94.8 ± 7.8	80.1 ± 2.7	69.3 ± 9.0	78.5 ± 6.6	11.4 ± 12.5	31.0 ± 2.9
δ^15^N	4.98 ± 1.68	1.27 ± 0.22	−1.43 ± 0.05	−1.28 ± 0.45	0.17 ± 0.44	−0.38 ± 0.14	1.54 ± 0.23	2.08 ± 0.21
Carbohydrates	24.3 ± 0.37	21.6 ± 1.45	28.0 ± 0.87	30.5 ± 3.26	25.4 ± 0.66	24.5 ± 1.42	29.0 ± 0.95	24.8 ± 2.1
Lipids and waxes	17.1 ± 0.94	21.7 ± 2.62	16.0 ± 0.64	15.8 ± 0.71	15.8 ± 0.24	16.2 ± 0.34	16.1 ± 0.10	16.6 ± 0.61
N-bearing compounds	27.4 ± 0.69	28.9 ± 0.27	21.7 ± 0.24	20.4 ± 0.90	28.6 ± 1.28	27.2 ± 0.32	29.6 ± 1.48	35.1 ± 1.61
aromatic compounds	9.55 ± 0.46	11.0 ± 1.11	6.22 ± 0.02	5.69 ± 0.32	6.85 ± 0.05	6.95 ± 0.36	8.96 ± 1.90	6.99 ± 0.21
Lignin	7.40 ± 0.61	4.75 ± 0.74	11.1 ± 0.35	13.0 ± 0.46	7.07 ± 0.05	8.07 ± 0.86	3.51 ± 0.42	2.73 ± 0.26
Phenolic compounds	14.3 ± 0.19	12.0 ± 0.93	17.1 ± 0.43	14.6 ± 1.48	16.3 ± 0.86	17.0 ± 0.39	12.8 ± 0.32	13.8 ± 0.23

**Table 2:** SOM chemistry of control and warming plots at 10–20 cm soil depth (n = 3). Values for carbohydrates, lipids and waxes, N-bearing compounds, aromatic compounds, lignin and phenolic compounds are derived from Py-GC/MS and are given in relative peak area. Bold numbers and asterisks indicate a statistically significant difference between control and warming treatments (* P-value < 0.05, ** P-value < 0.01). The plus and minus signs show whether warming caused an increase or a decrease in the respective parameter.

10–20 cm soil depth	Bulk		fPOM		oPOM		MaOM	
	Control	Warming	Control	Warming	Control	Warming	Control	Warming
% of bulk soil weight			2.35 ± 0.36	2.47 ± 0.16	22.6 ± 6.94	9.00 ± 2.20	69.1 ± 7.77	83.1 ± 3.15
Organic C content (% dry weight)	4.96 ± 0.75	4.74 ± 0.67	32.7 ± 0.58	30.0 ± 0.44	14.9 ± 0.44	15.6 ± 1.31	3.61 ± 0.58	3.65 ± 0.53
Total N content (% dry weight)	0.38 ± 0.05	0.34 ± 0.05	**1.08 ± 0.04**	0.96 ± 0.05 −**	0.98 ± 0.11	0.87 ± 0.05	0.33 ± 0.05	0.30 ± 0.05
C:N ratio	13.0 ± 0.28	13.7 ± 0.12	30.5 ± 0.71	31.5 ± 1.14	15.7 ± 1.31	18.0 ± 0.76	10.9 ± 0.59	12.1 ± 0.11
δ^13^C	−25.8 ± 0.07	−26.1 ± 0.05	−26.8 ± 0.21	−27.0 ± 0.31	−26.2 ± 0.17	−26.7 ± 0.14	**−25.6 ± 0.12**	−26.0 ± 0.07 −*
Δ^14^C	−11.1 ± 7.9	13.5 ± 6.6	57.3 ± 0.77	61.1 ± 7.1	23.4 ± 11.6	49.3 ± 3.3	−48.7 ± 12.6	−14.0 ± 8.6
δ^15^N	2.3 ± 0.26	2.03 ± 0.25	−0.6 ± 0.20	−0.73 ± 0.33	1.04 ± 0.39	0.12 ± 0.18	2.81 ± 0.17	2.19 ± 0.28
Carbohydrates	20.9 ± 0.77	19.0 ± 1.04	25.1 ± 0.58	26.3 ± 0.22	**37.4 ± 0.40**	25.6 ± 1.34 −**	24.8 ± 0.75	25.4 ± 0.75
Lipids and waxes	20.5 ± 0.46	22.8 ± 2.25	16.1 ± 0.51	17.0 ± 1.03	15.5 ± 0.76	16.2 ± 0.97	16.1 ± 0.18	15.7 ± 0.40
N-bearing compounds	33.5 ± 1.33	31.4 ± 1.34	22.0 ± 0.42	21.1 ± 0.50	24.1 ± 1.56	27.3 ± 0.33	**37.3 ± 0.28**	34.6 ± 0.59 −*
Aromatic compounds	8.75 ± 0.35	9.26 ± 0.73	7.03 ± 0.32	6.48 ± 0.14	6.27 ± 0.11	7.01 ± 0.23	7.84 ± 0.25	8.20 ± 0.43
Lignin	3.79 ± 0.29	4.46 ± 0.92	12.5 ± 0.47	12.9 ± 0.37	**3.27 ± 0.52**	6.93 ± 0.59 +*	2.78 ± 0.23	3.21 ± 0.25
Phenolic compounds	12.5 ± 0.92	13.1 ± 0.73	17.3 ± 0.17	16.4 ± 0.63	13.4 ± 1.24	16.9 ± 0.46	11.2 ± 0.92	12.9 ± 0.30

**Table 3:** Results of three-way-ANOVA of soil chemical parameters and permutational multivariate analysis of variance using distance matrices (ADONIS). ADONIS was performed with a Euclidean distance matrix including all 86 individual substances detected with Pyr-GC/MS. The same Euclidean distance matrix was used to create the NMDS plot in Fig. 3. Significant differences for individual factors (treatment, depth and fraction) as well as their interactions are indicated by asterisks.

	Treatment	Fraction	Depth	Treatment × fraction	Treatment × depth	Fraction × depth	Treatment × fraction × depth
Organic C content (% dry weight)		<0.001				0.010	0.035
Total N content (% dry weight)		<0.001					
C:N ratio		<0.001				0.014	
δ^13^C	0.026	<0.001				
Δ^14^C		<0.001	<0.001				
δ^15^N		<0.001	<0.001				
Carbohydrates				0.008		0.013	
Lipids and waxes							
N-bearing compounds		<0.001					0.021
Aromatic compounds		<0.001	0.036			0.023	
Lignin	0.041	<0.001					
Phenolic compounds		<0.001		0.027			

Chemical fingerprint (ADONIS)	<0.001	<0.001		0.005		<0.001	<0.001
