# Supplementary figures and images for: Little effects on soil organic matter chemistry of density fractions after seven years of forest soil warming
Source: Soil Biol Biochem. Author manuscript; Available in PMC 2016 Dec 29. (PMC5198888; doi:10.1016/j.soilbio.2016.09.003)

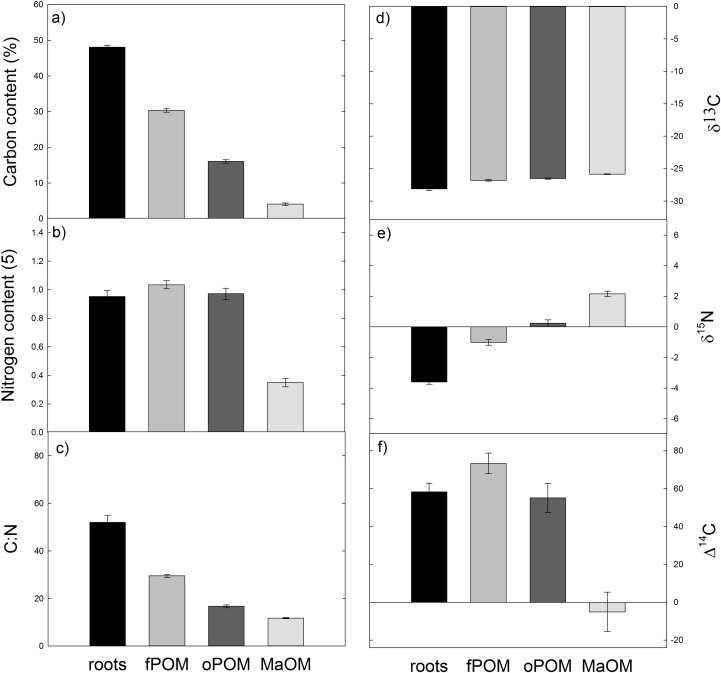

Supplement: FigS1 [file NIHMS70851-supplement-FigS1.jpg]

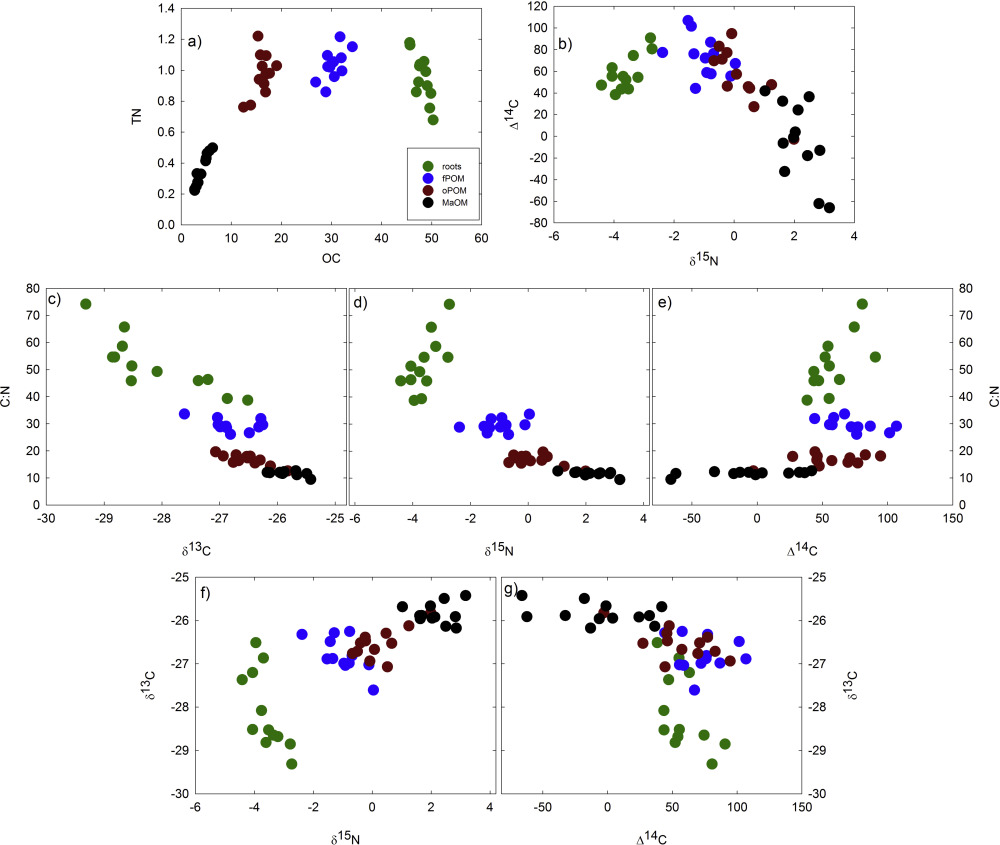

Supplement: FigS2 [file NIHMS70851-supplement-FigS2.jpg]
