# Supplementary material for: Little effects on soil organic matter chemistry of density fractions after seven years of forest soil warming
Source: Soil Biol Biochem. Author manuscript; Available in PMC 2016 Dec 29. (PMC5198888; doi:10.1016/j.soilbio.2016.09.003)
Supplement: TabS1 [file NIHMS70851-supplement-TabS1.docx]

Table S1. Compounds identified with Py-GC/MS, their species scores derived from NMDS and the mean species scores for the compound classes.

| retention time | substance | Compound Class | Species score 1 | Species score 2 |
| --- | --- | --- | --- | --- |
| 8.15 | Benzene, 1,3,5-trimethyl- | Aromatic | 0.0557 | 0.0055 |
| 8.35 | Styrene | Aromatic | 0.0360 | 0.0516 |
| 9.39 | Benzene, (2-methylpropyl)- | Aromatic | 0.0224 | 0.0161 |
| 9.72 | α-Methylstyrene | Aromatic | 0.0192 | 0.0185 |
| 12.55 | Indene | Aromatic | 0.0432 | 0.0359 |
| 15.47 | Acetophenone | Aromatic | 0.0272 | 0.0082 |
| 16.05 | Resorcinol | Aromatic | 0.0069 | 0.0031 |
| 19.75 | Benzene, (isocyanomethyl)- | Aromatic | 0.0223 | 0.0148 |
| 26.53 | 4-t-Butyl-o-xylene | Aromatic | -0.0772 | -0.0653 |
| 27.52 | Benzene, 1,2,4,5-tetramethyl- | Aromatic | -0.0376 | -0.0560 |
| 25.99 | Phthalan | Aromatic | -0.0222 | -0.0424 |
| 26.76 | Ethanone, 1-(2,4,6-trimethylphenyl)- | Aromatic | -0.0254 | -0.0242 |
| 10.47 | Furan, 2,5-dimethyl- | Carbohydrates | 0.0565 | 0.0367 |
| 11.45 | 3-Furaldehyde | Carbohydrates | 0.0279 | 0.0031 |
| 12.13 | 3-Furaldehyde | Carbohydrates | -0.0155 | -0.0009 |
| 12.88 | Benzofuran | Carbohydrates | 0.0059 | 0.0187 |
| 13.23 | Furan, 2,5-dimethyl- | Carbohydrates | 0.0183 | 0.0043 |
| 7.46 | 3-Cyclohexene-1-acetaldehyde, α,4-dimethyl- | Carbohydrates? | 0.0367 | 0.0601 |
| 8.85 | 2-Butanone, 3-hydroxy- | Carbohydrates? | 0.0042 | 0.0330 |
| 9.15 | 2-Propanone, 1-hydroxy- | Carbohydrates? | -0.0274 | -0.0286 |
| 11.61 | Cyclohexanone | Carbohydrates? | -0.0014 | -0.0034 |
| 11.97 | 2-Propanone, 1-hydroxy- | Carbohydrates? | 0.0029 | -0.0326 |
| 12.05 | Acetic acid | Carbohydrates? | -0.0425 | 0.0775 |
| 15.81 | Butanoic acid, 3-methyl- | Carbohydrates? | 0.0159 | 0.0195 |
| 15.91 | 3-Penten-2-one, 3-methyl- | Carbohydrates? | 0.0226 | 0.0164 |
| 17.33 | Cyclohexanone | Carbohydrates? | -0.0445 | -0.0372 |
| 20.64 | 2-Furanmethanol | Carbohydrates? | -0.0644 | -0.0396 |
| 25.16 | 2,5-Furandione, dihydro-3-methyl- | Carbohydrates? | 0.0140 | 0.0442 |
| 25.74 | 2,5-Furandione, dihydro-3-methyl- | Carbohydrates? | 0.0001 | 0.0057 |
| 18.69 | o-Guaiacol | Lignin | -0.0273 | -0.0486 |
| 20.14 | p-Methylguaiacol | Lignin | -0.0714 | -0.0767 |
| 21.2 | .p-Propylguaiacol | Lignin | -0.0448 | -0.0716 |
| 23.07 | Isoeugenol | Lignin | -0.0951 | -0.0973 |
| 25.44 | (E)-Isoeugenol | Lignin | -0.0918 | -0.0891 |
| 28.12 | Vanillin | Lignin | -0.0535 | -0.0567 |
| 28.95 | Acetoguaiacon | Lignin | -0.0359 | -0.0438 |
| 29.11 | p-Propylguaiacol | Lignin | -0.0633 | -0.0615 |
| 6.16 | Alkene (C11) | Lipids | 0.0461 | 0.0198 |
| 8.01 | Alkene (C13) | Lipids | 0.0251 | 0.0083 |
| 9.88 | Alkene | Lipids | 0.0262 | 0.0108 |
| 10.89 | Alkene | Lipids | 0.0137 | -0.0047 |
| 11.06 | Alkane | Lipids | 0.0035 | -0.0106 |
| 11.72 | Alkene | Lipids | 0.0234 | 0.0084 |
| 12.71 | Alkane | Lipids | 0.0265 | 0.0252 |
| 13.53 | Alkene | Lipids | -0.0027 | 0.0274 |
| 15.25 | Alkene | Lipids | 0.0019 | -0.0020 |
| 16.83 | Alkane | Lipids | 0.0414 | 0.0558 |
| 17.72 | Alkene | Lipids | 0.0112 | 0.0117 |
| 18.48 | Alkene | Lipids | -0.0086 | 0.0051 |
| 19.3 | Alkene | Lipids | 0.0222 | 0.0359 |
| 19.99 | Alkane | Lipids | 0.0041 | 0.0030 |
| 21.58 | Octanoic Acid | Lipids | -0.0146 | -0.0080 |
| 24.24 | Alkene | Lipids | -0.0668 | -0.0650 |
| 29.53 | C14H28O2 | Lipids | -0.0160 | 0.0059 |
| 16.32 | long chain alcohol | Lipids | 0.0069 | 0.0054 |
| 18.21 | 2-Cyclopenten-1-one, 2-hydroxy-3-methyl- | Lipids | -0.0178 | -0.0236 |
| 18.62 | long chain alcohol | Lipids | -0.0192 | -0.0268 |
| 24.53 | long chain alcohol | Lipids | 0.0199 | 0.0025 |
| 6.94 | Pyridine | Proteins | 0.0513 | -0.0016 |
| 7.58 | Pyridine, 2-methyl- | Proteins | 0.0551 | 0.0470 |
| 8.49 | Pyrimidine, 4-methyl- | Proteins | 0.0314 | 0.0125 |
| 9.03 | Pyridine, 3-methyl | Proteins | 0.0619 | 0.0085 |
| 10.22 | 1H-Imidazole, 1-methyl- | Proteins | 0.0381 | 0.0212 |
| 12.96 | Pyrrole | Proteins | 0.0499 | 0.0091 |
| 13.6 | 1H-Pyrazole, 1,3,5-trimethyl- | Proteins | 0.0021 | 0.0460 |
| 13.65 | 1,3-Diazine | Proteins | 0.0675 | -0.0090 |
| 13.93 | 1,3-Diazine | Proteins | 0.0415 | -0.0051 |
| 14.12 | 1H-Pyrazole, 1,3,5-trimethyl- | Proteins | -0.0263 | -0.0007 |
| 14.47 | 4-Pyridinamine | Proteins | 0.0450 | 0.0090 |
| 15.52 | 4-Pyridinamine | Proteins | 0.0767 | 0.0247 |
| 17.1 | Acetamide | Proteins | 0.0051 | 0.0123 |
| 18.86 | 2,4-Imidazolidinedione, 1-methyl- | Proteins | -0.1136 | -0.0736 |
| 19.45 | Caprolactam | Proteins | 0.0342 | 0.0468 |
| 21.13 | 2(1H)-Pyridinone | Proteins | 0.0293 | 0.0082 |
| 21.4 | Thiocyanic acid, phenylmethyl ester | Proteins | 0.0341 | 0.0257 |
| 26.61 | Indole | Proteins | -0.0052 | -0.0122 |
| 26.88 | Cyclohexanamine | Proteins | 0.0208 | 0.0384 |
| 27.17 | 1H-Indole, 3-methyl- | Proteins | 0.0231 | 0.0130 |
| 27.3 | m-Nitroaniline | Proteins | -0.0294 | -0.0138 |
| 20.79 | Phenol, 3-methyl- | Phenol | 0.0044 | -0.0099 |
| 20.83 | Phenol | Phenol | 0.0068 | 0.0017 |
| 21.74 | Phenol, 3-ethyl- | Phenol | 0.0017 | -0.0057 |
| 21.91 | Phenol, 4-methyl- | Phenol | -0.0102 | -0.0211 |
| 22.03 | Phenol, 3-methyl- | Phenol | 0.0007 | -0.0220 |
| 23.18 | Phenol, 4-ethyl- | Phenol | 0.0120 | -0.0038 |
| 23.48 | Phenol, p-tert-butyl- | Phenol | -0.0771 | -0.0855 |
|  |  |  |  |  |
|  |  | Aromatic | 0.0059 | -0.0029 |
|  |  | Carbohydrates | 0.0005 | 0.0104 |
|  |  | Lignin | -0.0604 | -0.0682 |
|  |  | Lipids | 0.0060 | 0.0040 |
|  |  | Proteins | 0.0234 | 0.0098 |
|  |  | Phenol | -0.0088 | -0.0209 |
