# Supplementary material for: Little effects on soil organic matter chemistry of density fractions after seven years of forest soil warming
Source: Soil Biol Biochem. Author manuscript; Available in PMC 2016 Dec 29. (PMC5198888; doi:10.1016/j.soilbio.2016.09.003)
Supplement: TabS2 [file NIHMS70851-supplement-TabS2.docx]

Table S2: Correlation of warming induced changes across all three density fractions (calculated by subtracting values for warming treatment from control values). Bold numbers indicate significant correlation coefficients.

|  | ΔOC | ΔTN | ΔC:N | Δδ^13^C | Δ∆^14^C | Δδ^15^N |
| --- | --- | --- | --- | --- | --- | --- |
| ΔOC | **** | **0.84** |  |  |  |  |
| ΔTN |  | **** |  |  |  |  |
| ΔC:N |  |  | **** | **-0.53** | **0.43** |  |
| Δδ^13^C |  |  |  | **** | **-0.44** |  |
| Δ∆^14^C |  |  |  |  | **** |  |
| Δδ^15^N |  |  |  |  |  | **** |
