# Supplementary material for: Little effects on soil organic matter chemistry of density fractions after seven years of forest soil warming
Source: Soil Biol Biochem. Author manuscript; Available in PMC 2016 Dec 29. (PMC5198888; doi:10.1016/j.soilbio.2016.09.003)
Supplement: TabS3 [file NIHMS70851-supplement-TabS3.docx]

| Pyr x | all | fPOM | oPOM | MaOM |
| --- | --- | --- | --- | --- |
| OC | **0.19** | n.s. | n.s. | n.s. |
| TN | **0.18** | n.s. | n.s. | n.s. |
| C:N | **0.18** | n.s. | **0.12** | n.s. |
| δ^13^C | **0.07** | n.s. | n.s. | n.s. |
| ∆^14^C | **0.06** | n.s. | **0.18** | n.s. |
| δ^15^N | **0.19** | n.s. | n.s. | n.s. |

Table S3: Mantel test of Pyrolysis distance matrix and soil parameters. Bold numbers are significant R².
